# Supplementary material for: Improved characterisation of MRSA transmission using within-host bacterial sequence diversity
Source: eLife. 2019 Oct 8;8:e46402. doi: 10.7554/eLife.46402 (PMC6954020; doi:10.7554/eLife.46402)
Supplement: Supplementary file 1. — Given are the values of the statistic and p-values estimated by permuting the tip states 1000 times. (A) Blue tips occur only in one clade but that clade is not exclusively blue. The AI statistic shows significant deviation from the null hypothesis of no phylogeny-state association, but the others do not. (B) Blue tips form a single clade. All statistics other than the MC for the red state show significant deviation from the null. (C) Blue tips appear randomly in the phylogeny. There is no evidence of any association using any statistic. (D) A single blue tip is basal to the remainder of the tree. The AI statistic and the MC for the red state suggest an association. (E/F) A single blue tip occurs amongst the red tips. Some evidence of association may still be identified using the AI statistic and further investigation is warranted. In cases D-F the PS and MC (blue) statistics are always equal to 1. [file elife-46402-supp1.docx]

**Supplementary file 1:** Examples of arrangements of tips with two states (red and blue) on a phylogeny and the corresponding values of the AI, PS, and MC statistics. Given are the values of the statistic and a *p*-values estimated by permuting the tip states 1000 times. A) Blue tips occur only in one clade but that clade is not exclusively blue. The AI statistic shows significant deviation from the null hypothesis of no phylogeny-state association, but the others do not. B) Blue tips form a single clade. All statistics other than the MC for the red state show significant deviation from the null. C) Blue tips appear randomly in the phylogeny. There is no evidence of any association using any statistic. D) A single blue tip is basal to the remainder of the tree. The AI statistic and the MC for the *red* state suggest an association. E/F) A single blue tip occurs amongst the red tips. Some evidence of association may still be identified using the AI statistic and further investigation is warranted. In cases D-F the PS and MC (blue) statistics are always equal to 1.
