# Supplementary material for: Improved characterisation of MRSA transmission using within-host bacterial sequence diversity
Source: eLife. 2019 Oct 8;8:e46402. doi: 10.7554/eLife.46402 (PMC6954020; doi:10.7554/eLife.46402)
Supplement: Supplementary file 2. [file elife-46402-supp2.docx]

| **Start position** | **End position** | **Feature** |
| --- | --- | --- |
| 34122 | 67172 | SCCmercury |
| 67173 | 102467 | SCCmec type III |
| 376406 | 419745 | prophage phiSa1(TW20) |
| 486820 | 518088 | putative transposon/ICE Tn5801 |
| 952129 | 966671 | putative pathogenicity island |
| 1228484 | 1229807 | IS256 |
| 1401705 | 1403028 | IS256 |
| 1516700 | 1517444 | IS200-family |
| 1723773 | 1725096 | IS256 |
| 1775192 | 1781878 | Transposon Tn554 |
| 1913820 | 1915143 | IS256 |
| 1940775 | 1941519 | IS200-family |
| 1955681 | 1957003 | IS256 |
| 2017327 | 2018838 | IS1181 |
| 2108459 | 2153126 | Prophage phiSa3(TW20) |
| 2181686 | 2308888 | Prophage phiSa5(TW20) |
| 2442871 | 2444383 | IS1181 |
| 2873696 | 2893550 | integrative conjugative element ICE6013 |
| 3036502 | 3037246 | IS200-family |

**Supplementary file 2:** Coordinates of the accessory regions of the TW20 chromosome.
