# Supplementary material for: Improved characterisation of MRSA transmission using within-host bacterial sequence diversity
Source: eLife. 2019 Oct 8;8:e46402. doi: 10.7554/eLife.46402 (PMC6954020; doi:10.7554/eLife.46402)
Supplement: Supplementary file 3. — Positions are with respect to the TW20 reference strain. [file elife-46402-supp3.docx]

| **Start position** | **End position** |
| --- | --- |
| 531528 | 532588 |
| 681826 | 688849 |
| 692479 | 692596 |
| 742747 | 742791 |
| 858452 | 858480 |
| 931230 | 931296 |
| 931374 | 931632 |
| 931886 | 931917 |
| 2019055 | 2019079 |
| 2168835 | 2168952 |
| 2828633 | 2828718 |
| 2941948 | 2942008 |
| 2942026 | 2942062 |
| 2942128 | 2942164 |
| 2981147 | 2981273 |

**Supplementary file 3:** Coordinates of regions of the MRSA genome in which recombination was identified by Gubbins. Positions are with respect to the TW20 reference strain.
